# Ethical Practices for Health Research in the Eastern Mediterranean Region of the World Health Organization: A Retrospective Data Analysis

**DOI:** 10.1371/journal.pone.0002094

**Published:** 2008-05-07

**Authors:** Mohammad Abdur Rab, Mohammad Afzal, Alaa Abou-Zeid, Henry Silverman

**Affiliations:** 1 Country Office, World Health Organization (WHO), Khartoum, Sudan; 2 Eastern Mediterranean Regional Office, Research Policy and Cooperation Unit, World Health Organization (WHO), Cairo, Egypt; 3 University of Maryland School of Medicine, Baltimore, Maryland, United States of America; London School of Hygiene and Tropical Medicine, Peru

## Abstract

**Background:**

Commentators have expressed concern regarding the existence of proper ethics review systems in developing countries. Our aim is to explore the extent with which investigators from countries in the Eastern Mediterranean (EM) Region consider several ethical practices in the conduct of their research.

**Methodology/Principal Findings:**

Investigators from 12 countries in the EM region submitted 143 proposals involving Public Health and Biotechnology & Genomics to a grant scheme funded by the Eastern Mediterranean Regional Office of the WHO and the Organization of Islamic Conference Standing Committee for Science and Technological Cooperation in 2006. The grant application included a 1-page questionnaire that asked investigators 1) whether ethical clearance was obtained, 2) whether they plan to obtain informed consent, and 3) whether confidentiality of human subject data would be ensured. The methodologies of the submitted researches were categorized as to whether it involved 1) human subject research (e.g., the prospective collection of biological specimens or the performance of qualitative research), 2) research that could be exempt from ongoing ethics review, and 3) research not involving human subjects. A descriptive analysis was used to analyze the investigators' responses and a chi-square analysis was used to analyze categorical variables. Of the 79 submitted proposals determined to involve “human subjects”, ethical clearance was not obtained in 29%; investigators thought that informed consent was not needed in 29%; and investigators did not mention that they would ensure confidentiality of the obtained data in 8% of the studies. The magnitude of these deficiencies was similar regardless of study design type, i.e., prospective collection of biological samples and qualitative research methods.

**Conclusion/Significance:**

These results suggest that attention to ethical safeguards is not optimal among investigators in the EM Region. Further guidelines for strengthening ethical review systems, as well as enhanced educational training in concepts of research ethics for investigators are warranted in this region.

## Introduction

Coinciding with the recent increase in the conduct of research involving human subjects in developing countries there has been the development of several guidelines, regulations, and recommendations regarding the ethics of health research [1]–[3]. Despite the existence of such documents, concern has been expressed regarding the presence and adequacy of ethics review systems in health research in developing countries, including those from the Eastern Mediterranean (EM) Region [4], 5. Another issue is the extent to which investigators are aware of the ethical considerations of their research. Little empirical research has been conducted on the research ethics practices of investigators in the developing world. Several papers have described in qualitative terms the status of ethics review systems and the awareness and practices of investigators regarding health research ethics in developing countries [6]–[8]. Two studies have explored the extent to which investigators obtain ethics review. One study involving researchers from Asia, Africa, and South America revealed that 25% of the investigators reported that their studies did not undergo ethics review [5]. The other study showed that among investigators who submitted research proposals funded by the Eastern Mediterranean Regional Office in 2003, 43% felt their research did not require ethical clearance [9]. A clearer picture regarding the extent to which investigators consider the ethical aspects of their research can help assess the ethics training needs of health researchers and provide further impetus to policymakers to strengthen ethical review systems in the EM Region. Accordingly, the purpose of this study was to analyze the ethical practices undertaken by health researchers from the EM Region who had submitted proposals for funding in 2006. We demonstrate in this larger study that the ethical practices of investigators in the EM Region regarding attaining ethical clearance, plans for obtaining informed consent, and ensuring confidentiality of sensitive data needs improvement.

## Methods

### Participants/Setting

In 2006, the Eastern Mediterranean Regional Office (EMRO) of the WHO and the Organization of Islamic Conference Standing Committee for Science and Technological Cooperation (COMSTECH) jointly supported a grant scheme in the priority areas of public health and applied biotechnology & genomics. Applications were accepted from any of the 22 countries within the Eastern Mediterranean (EM) Region; these included: Afghanistan, Islamic Republic of Iran, Pakistan, Iraq, Jordan, Lebanon, Syrian Arab Republic, Palestine, Morocco, Tunisia, Libya, Egypt, Sudan, Somalia, Djibouti, the Sultanate of Oman, Kingdom of Saudi Arabia, United Arab Emirates, Qatar, Kuwait, Yemen, and Bahrain.

A one-page questionnaire was included in the grant application, to which investigators were to provide information regarding certain ethical practices of their research. Specifically, the questionnaire asked the applicants to answer the following questions: a) “Has ethical clearance been obtained for the conduct of the study?” and “If ethical clearance not obtained, please state reasons”; b) “Shall informed consent be obtained from the human subjects?” (and “Please attach consent letter(s)/forms(s)”); and c) “Shall confidentiality of participants be protected?” The investigators' responses to this one-page questionnaire are the subject of this study's analysis.

### Description of Procedures

To obtain a better reflection of the appropriateness of the ethical practices of investigators, we sought to determine the extent to which the ethical practices were relevant to the submitted proposals. For example, research that meets a regulatory definition “exempt” research or “non-human subject research” might not require full ethics committee review, informed consent or confidentiality protections. The sponsors of the grant scheme did not give explicit guidelines regarding the definitions of exempt and non-human subject research and only three of the 12 countries from where proposals were submitted have national regulations regarding research ethics. Accordingly, submitted proposals were categorized by using the definitions in the U.S. Federal Regulations regarding exempt research and non-human subject research [10]. One of the authors (HJS) reviewed the methodology of all of the proposals and used the following decision tree to categorize the proposals:

First, it was determined whether the study met the definition of human subject research according to the following definition [10]:

*Research* means a systematic investigation, including research development, testing and evaluation, designed to develop or contribute to generalizable knowledge.

*Human subject* means a living individual about whom an investigator (whether professional or student) conducting research obtains (1) Data through intervention or interaction with the individual, or (2) Identifiable, private information.

*Intervention* includes both physical procedures by which data are gathered (for example, venipuncture) and manipulations of the subject or the subject's environment that are performed for research purposes. Interaction includes communication or interpersonal contact between investigator and subject. *Private information* includes information about behavior that occurs in a context in which an individual can reasonably expect that no observation or recording is taking place, and information which has been provided for specific purposes by an individual and which the individual can reasonably expect will not be made public (for example, a medical record). Private information must be individually identifiable (i.e., the identity of the subject is or may readily be ascertained by the investigator or associated with the information) in order for obtaining the information to constitute research involving human subjects.

For proposals meeting the definition of human subject research, it was then determined which of the proposals would fit one of the following exemption categories adapted from the U.S. regulations [10]:

1. **Normal educational practices:**
  - Research takes place entirely within an established or commonly accepted educational setting (i.e., within officially recognized school or training program); and
  - Research involves only normal educational practices (i.e., instructional strategies or techniques, curricula); and
  - There are not any other elements to the research study (beyond educational practices).
2. **Questionnaires, interviews, and focus group discussions:**
  - The research is limited to educational tests, survey procedures, interview procedures, or observation of public behavior (no other data); and EITHER
    - The information obtained is recorded in such a way that human subjects cannot be identified (directly or through identifiers or through codes) – OR –
    - While the information obtained is identified or coded, disclosure of the human subjects' responses outside the research would not reasonably place the subjects at risk of criminal or civil liability, or be damaging to the subjects' financial standing, employability, or reputation.
3. **Chart review and biological specimen studies:**
  Research involving the collection or study of EXISTING data, documents, records, pathological specimens, or diagnostic specimens, if these sources are publicly available or if the information is recorded by the investigator in such a manner that subjects cannot be identified, directly or through identifiers linked to the subjects.

If one of the exemption categories was applicable to the proposal, it was then determined whether the research, nonetheless, would not be considered exempt; for example, if there were plans to enroll children or other vulnerable subjects, or if the risk to human subjects from answering questions in qualitative studies would be above minimal risk (e.g., if questionnaires contain invasive questions that may cause the subject to experience emotional distress or discomfort while answering them, in other words, the potential risks of the research may negate the exemption, because the research could be determined to be above minimal risk). Finally, it was determined if any of the proposals would fulfill the criteria of the U.S. regulations for a waiver of informed consent [10].

Proposals submitted for funding involved several different types of research methods, e.g., analysis of prospectively obtained biological specimens, qualitative research, analysis of archived data, and receipt of previously collected de-identified biological specimens. The prospective obtainment of biological samples from subjects involves an *intervention* with human subjects that differ in kind from the *interaction* that occurs with qualitative research designs (e.g., use of questionnaires, interviews, and focus group discussions), and hence, investigators might hold different perceptions regarding the need for certain ethical safeguards. Accordingly, we categorized the proposals as to whether it involved prospective collection of biological samples or the use of qualitative methods. Qualitative studies were further categorized as to whether a social behavioral intervention was part of the design (e.g., counseling, training, or educational intervention) or whether it involved only the use of questionnaires, interviews, or focus group discussions.

Another issue we became aware was that it was not unusual for many proposals at the time of application to be either under ethics committee review or that there would be plans to submit proposals to an ethics review committee in the near future. Such a “pending” category might be different from proposals that had obtained ethics clearance, as well as different from proposals that had not indicated it had obtained ethical clearance. Accordingly, we decided to show this “pending” category in our analysis. We also collected information regarding whether investigators responded by stating “yes”, “no”, or “no answer”. We also reviewed, when available, the reasons the investigators stated for not obtaining ethical clearance for their proposals.

To determine the extent of variation of ethical considerations between countries, we analyzed aggregate data for the countries that submitted five or more proposals. We searched various databases, e.g., United Nations Educational, Scientific and Cultural Organization (UNESCO), World Health Organization (WHO), and Office of Human Research Protections (OHRP), regarding the existence of national research ethics regulations/guidelines and national ethics committees in order to determine whether countries with such regulations/guidelines were more likely to have researchers who adhered to generally accepted ethical practices.

### Ethics

The study was reviewed by the institutional review board at the University of Maryland School of Medicine. Informed consent was not obtained, because de-identified data were analyzed.

### Statistical Methods

A chi-square analysis was used for comparisons between categorical variables.

## Results

Health researchers from 12/22 (55%) countries within the EM Region submitted a total of 143 applications. These countries included: Iran, Egypt, Jordan, Lebanon, Morocco, Oman, Pakistan, Sudan, Syrian Arab Republic, Kuwait, Yemen, and Tunisia. Of these applications, 20/143 (14%) involved animal research and 15/143 (10%) involved literature review, analysis of health systems outcome data, or analysis of non-human specimens (e.g., water analysis or vector research). The remaining 108/143 (76%) proposals constituted the subject of our analysis.

Proposals were categorized as follows (see Table 1): analysis of prospectively collected biological samples; qualitative research involving a social behavioral intervention and the use of either questionnaires, interviews, or focus group discussions; qualitative research involving either questionnaires, interviews, or focus group discussions; exempt research (either qualitative research or analysis of archived data recorded without identifiers), and non-human subjects research (receipt of existing biological specimens that were de-identified). None of the qualitative studies involved normal educational practices. Seventeen qualitative research studies involving questionnaires, interviews, or focus groups discussions did not qualify for exempt status based on the following criteria: a) the obtainment of identifiable information that could be damaging to the subject's employability or reputation (n = 10), b) the inclusion of questions on surveys or interviews that might evoke greater than minimal risk emotional responses (n = 3), or c) involvement of children or adolescents less than 18 years of age (n = 9); several studies met more than one of these criteria. Twenty research studies involving qualitative methods qualified for exempt status.

**Table 1:** Ethical considerations in submitted proposals (N = 108).

Type of Research	Proposals	Ethical Clearance			Informed Consent	Confidentiality
		Obtained	Pending	Total		
	N	N	N	N (%)	N (%)	N (%)
**HUMAN SUBJECT RESEARCH**						
**Biological Samples**	49	28	8	36 (74)	37 (76)	43 (88)
**Qualitative Research involving an intervention**	13	5	5	10 (77)	8 (62)	13 (100)
**Qualitative Research: Questionnaires, interviews, or focus group discussions**	17	5	5	10 (59)	11 (65)	17 (100)
**Total**	**79**	**38**	**18**	**56 (71)**	**56 (71)**	**73 (92)**
**EXEMPT RESEARCH**						
**Qualitative Research: Questionnaires, interviews, or focus group discussions**	20	9	2	10 (50)	10 (50)	15 (75)
**Collection of archived data/pathologic specimens (recorded without identifiers)**	3	0	0	0 (0)	0 (0)	2 (67)
**Total**	**23**	**9**	**2**	**10 (43)**	**10 (43)**	**17 (74)**
**NON-HUMAN SUBJECT RESEARCH**						
**Receipt of de-identified biological samples**	**6**	**0**	**1**	**1 (17)**	**0 (0)**	**2 (33)**
**Grand Total**	**108**	**47**	**21**	**67 (62)**	**66 (61)**	**92 (85)**

Table 1 shows the extent to which investigators considered the various ethical safeguards categorized according to the type of research design. Results for the three research ethics practices (ethical clearance, informed consent, and confidentiality) were similar among the different non-exempt researches involving human subjects (p>0.05). These results were higher than those observed for proposals categorized as exempt or non-human subjects research (p<0.003). Regarding the 50 qualitative researches (both exempt and non-exempt), 29 investigators stated they would obtain informed consent, 15 stated they would not obtain informed consent, and 6 gave no answer. Of the 15 qualitative proposals in which investigators stated they would not obtain informed consent, 7 included interviews or focus groups discussions, while the other 8 involved only the use of questionnaires.

Table 2 shows the aggregate data for ethical practices for the four countries (shown anonymously) that submitted 5 or more proposals. For the non-exempt human subject researches, no significant differences were observed in the extent to which the ethical practices were considered between these four countries (p>0.05). Ethical clearance (obtained or pending) for proposals involving human subjects ranged between 62 and 100%; the plan to obtain informed consent ranged between 62 and 88%; and the plan to ensure the confidentiality of data ranged between 77–100%. Of these countries, only Country A has national regulations addressing the ethics of research practices. All four of these countries have national ethics committees. These results are similar to the overall aggregate data for all countries. Regarding the existence of national ethics committees for all 12 countries from where proposals were submitted, 10 have such committees and three are known to have national regulations/guidelines addressing the ethics of research.

**Table 2:** Ethical considerations in proposals from countries that submitted five or more proposals.

Type of Research	Proposals (N)	Ethical Clearance N (%)	Informed Consent N (%)	Confidentiality N (%)
**COUNTRY A**				
Biological Samples	22	17	19	19
Qualitative research involving an intervention	5	2	2	5
Qualitative: Questionnaires, interviews, focus group discussions	7	2	1	7
**TOTAL HUMAN SUBJECT RESEARCH**	**34**	**21 (62)**	**22 (65)**	**32 (94)**
Exempt Research	6	3	3	5
Non-Human Subject Research	2	0	0	0
**COUNTRY B**				
Biological Samples	8	6	6	8
Qualitative research involving an intervention	1	1	1	1
Qualitative: Questionnaires, interviews, focus group discussions	7	7	7	7
**TOTAL - HUMAN SUBJECT RESEARCH**	**16**	**14 (88)**	**14 (88)**	**16 (100)**
Exempt Research	3	1	0	0
Non-Human Subject Research	0	0	0	0
**COUNTRY C**				
Biological Samples	9	6	5	6
Qualitative research involving an intervention	4	3	3	4
Qualitative: Questionnaires, interviews, focus group discussions	0	0	0	0
**TOTAL - HUMAN SUBJECT RESEARCH**	**13**	**9 (69)**	**8 (62)**	**10 (77)**
Exempt Research	3	1	1	1
Non-Human Subject Research	4	2	0	2
**COUNTRY D**				
Biological Samples	3	3	2	3
Qualitative research involving an intervention	2	2	1	2
Qualitative: Questionnaires, interviews, focus group discussions	0	0	0	0
**TOTAL - HUMAN SUBJECT RESEARCH**	**5**	**5 (100)**	**3 (60)**	**5 (100)**
Exempt Research	2	0	2	2
Non-Human Subjects Research	0	0	0	0

Table 3 shows a summary of the reasons given by investigators for not obtaining ethical clearance categorized according to the type of research. Several investigators felt that obtaining biological samples from patients seeking medical care did not require ethical clearance. Many investigators thought that research involving qualitative research does not require ethical clearance, because it does not involve an intervention, an invasive procedure, or the administration of drugs. Also, several investigators planning to do qualitative research involving children did not think ethical clearance was necessary. In all types of research studies, several investigators thought that approval from other entities (e.g., ministry of health, director of the hospital, and university internal review board) could substitute for a review by an independent ethics review committee.

**Table 3:** Reasons given by investigators as to why ethical clearance was not obtained (n = 25).

Type of Research	Reason stated for not obtaining ethical clearance
**Biological Samples (n = 6)**	*“biological samples will be obtained from patients who will be seeking medical care”*
	“*Authorization letter will be obtained from the ministry of health”*
	*“Ethical approval will be obtained by my collaborator as I will not directly deal with human beings.”* (no indication was given as to whether the samples would be de-identified when transferred to the investigator)
	*“[ethics committee] not concerned with this project*.” (project involves skin biopsies obtained prospectively)
**Qualitative Research: Social Behavioral Intervention with questionnaires, interviews, or focus group discussions (n = 3)**	“*ethics committee not formed, permission obtained from university internal review board”*
	“*not necessary because the data collected are not confidential*” (study involves participation of adolescents
	“[permission will be] *obtained from the director of the hospital*”
**Qualitative Research: Questionnaires, interviews, or focus group discussions (n = 7)**	“*information in questionnaire remains secret, mothers are not forced to take the counseling*” (study involves the effect of a counseling on maternal mental health)
	“*will not involve any intervention or invasive procedures*” (survey of adolescents regarding the use of illicit drugs);
	“*not necessary because the data collected are not confidential*” (study involves participation of children);
	*“scientific committee at the faculty of nursing[will review the research]”* (50) (study involves the collection of mental health data from adolescents)
	“This *study is anonymous and has no treatment or intervention….risks of this research study are minimal….participant might experience some psychological discomfort while completing the forms*. (Study involves adolescents; mental health data will be collected that could be damaging to reputation, and data will be coded and hence, not anonymous).
**Exempt Qualitative Research (n = 5)**	“there is no intervention, no ethical approval has been obtained (collection of anonymous, non-damaging data)
	“it is not necessary, ethical considerations for doing this research (study involves opinions of staff regarding management systems information)
	“as we will not do any invasive procedures, there is no need to assess safeguards except notice to confidentiality of Focus Group Discussions and questionnaire” (study involves survey of communities involving disaster plan management)
	“*study does not involve testing or drugs, etc, that comes under ethical consideration, therefore it does not apply”* (study involves collecting quality of life data from patients)
	“*The current research will neither include patient's data nor trial of medication*” (study involves the anonymous opinions of the hospital staff regarding patient access to health care services)
**Exempt Research: Analysis of archived data recorded without identifiers (n = 2)**	“*The proposed project is a retrospective study. Only archived biopsies will be used….epidemiologic and clinical data will be obtained in an anonymous fashion*.”
**Non-Human Subjects Research: Receipt of existing de-identified biological samples (n = 2)**	“*will do in-vitro research on samples obtained from patients*” (protocol described that samples will not have any patient identifying data)

## Discussion

Health research can play a crucial role in improving national and global health by developing and evaluating interventions and by exploring strategies that can empower individuals to alter unhealthy behaviors. However, health research involves human subjects and such individuals might be harmed by their participation in research. Accordingly, a strong system of ethical review is needed to enhance the protections of the rights and welfare of human subjects. Also, to enhance the public trust in research activities, investigators need to subscribe to a strict code of ethics that equals the highest standard of respect for human rights. This framework thus places ethics at the very core of a country's programs for health and development [11].

The present study shows that, in general, the extent to which investigators in the EM Region consider ethics safeguards in the conduct of their research requires improvement. Of the submitted proposals determined to involve “human subjects”, ethical clearance was not obtained in 29% of the proposals; investigators thought that informed consent was not needed in 29% of the submitted studies; and investigators did not mention any measures to ensure confidentiality of the obtained data in 8% of the studies. The magnitude of these deficiencies was similar regardless of study design type, i.e., prospective collection of biological samples and qualitative research methods. Our results are made more significant by the fact that the summary data regarding ethical practices excluded research that could be determined by a research ethics committee to be either exempt from their ongoing review or not to involve human subjects. Indeed, percentages for the ethical practices observed for these types of researches were lower compared to the non-exempt human subject research.

It is not clear why 15 of 50 investigators planning to do qualitative research (exempt and non-exempt research) stated they would not obtain informed consent. Obviously, subjects who complete questionnaires or participate in interviews or focus group discussions are giving their consent to a certain extent, even if they might not be fully informed. To account for these responses, investigators might have believed that informed consent was not practicable (six studies planned to enroll “hundreds” of subjects to complete questionnaires) or that they were not required to give subjects a full explanation of the research due to the setting of the research (e.g., six studies planned to involve students or staff of an organizations). Alternatively, investigators might have thought they were being asked if they planned to obtain written informed consent, as the one-page questionnaire of the grant application asked investigators to “please attach the consent letter”.

A review of the reasons given for not obtaining ethical clearance revealed that many researchers thought that research involving questionnaires, interview, or focus group discussions do not require ethical clearance, because such research does not involve an intervention or the administration of experimental drugs. This finding might reflect a lack of understanding of the psychological and social harms that can occur from obtaining private and sensitive data. Also, several investigators did not consider a need for additional ethical safeguards for research involving the participation of adolescents. Finally, a few investigators felt that ethical clearance and informed consent were not necessary when obtaining biological samples from patients seeking medical care. Such an attitude might be due to several perspectives; for example, investigators might believe that such patients, especially those in the public sector, might have an obligation to participate in research. Additionally, investigators might think that obtaining “left-over” samples of biological tissues involves no additional risk to the patients and hence, do not require informed consent or ethical clearance from an independent committee. However, regardless of the risk of the study, failure to obtain informed consent is not respectful of human subject rights. Also, all research ethics guidelines recommend independent review of all human subject research [2], [3], [10].

Two other studies have reported on the extent to which ethical review is obtained in developing countries. Hyder and colleagues reported the results of a self-administered survey that asked investigators from Asia, African, and South American questions regarding a previous research project [5]. This study observed that 25% of the investigators stated that their studies did not undergo some form of ethics review. Another study involving investigators from the Eastern Mediterranean Region who submitted proposals for funding to EMRO/WHO in 2003 revealed that 43% of the investigators did not believe that their proposals required ethical clearance [9]. This previous study did not indicate whether exempt and non-human subject researches were excluded from the analysis and hence, it is difficult to compare these results to those obtained in the present study.

Several factors might account for the present findings. First, failure to obtain ethical clearance might be indicative of the absence of a system of ethical review at the investigators' respective institutions. Second, investigators might be unaware of the need for independent ethical clearance, informed consent, and confidentiality protections. Lack of awareness might result from the failure of the undergraduate and post-graduate curriculum to include materials in the area of research ethics. Also, unawareness might result from the lack of support for research ethics practices at the national level. Indeed, of the 12 countries represented in this study, only three are known to have national regulations mandating the existence of research ethics committees and the need for informed consent. To be sure, 10 of the 12 countries represented in the present sample are known to have national ethics committees. National ethics committees might be involved in setting ethics standards, review of national research, or providing ethics education and any one of these activities could be expected to influence the practices of investigators. However, our findings might indicate the relative failure of national regulations or national ethics committees to have a downstream effect on the practices of the research staff. The lack of guidance at the national level might also lead to inconsistent approaches among investigators and academic officials regarding the types of researches that might be categorized as exempt or as non-human subject research.

Key limitations of the present study include the limited scope of the types of the research proposals reviewed and hence, the generalizability of the observed results might be limited. For example, the proposals analyzed in the present study represent the limited priority areas of public health and biotechnology and genomics and therefore, might underestimate the practices of investigators performing pharmaceutical-sponsored research that usually require ethical review, informed consent, and confidentiality assurances. On the other hand, the research proposals represent those submitted to a highly competitive WHO/COMSTECH grant scheme and therefore, reflect the practices of senior investigators who might be expected to be aware of ethical practices regarding research. Accordingly, our results might overestimate the practices of more junior investigators and those in training who submit research proposals for thesis projects. Also, our data regarding the obtainment of ethics review included the intent of investigators to obtain such review. Since we do not have information as to whether such review occurred, our results might overestimate the extent to which ethics review was obtained. Finally, scientific review committees in the absence of research ethics committees might have been involved in reviewing the ethical aspects of the submitted protocols. Accordingly, investigators might have indicated that ethical clearance was obtained if their proposals received review from such scientific review committees. However, the WHO states in their guidelines that ethics review should be separate from the scientific review of research [7]. Having said this, we are not aware as to whether local scientific review was a requirement in any of the countries in the EM region.

Another limitation of the study stems from the use of criteria in the U.S. federal regulations to categorize studies as being exempt or non-human subject research. The use of such criteria might not be relevant to the local context or might differ from those embraced by national authorities or international funders. In the absence of explicit, external guidelines, investigators might have taken upon themselves to determine the types of research that could be classified as exempt or non-human subject research and accordingly, decide by themselves which proposals require ethical clearance, informed consent, and the protection of confidential information. Finally, our data relied heavily on the self-report of investigators and therefore, bias might have been introduced by the motivation of the investigators to obtain funding.

Our results have several implications for research and research ethics in the Eastern Mediterranean Region. First, the lack of a firm foundation and affirmation for research ethics might impact negatively on the ability of investigators to obtain internationally funded research. Indeed, many funders (both private and governmental) from the U.S., Europe, and Canada have stringent research ethics requirements that might be met more readily if national regulations exist or if a national ethics committee conduct overview of such research. Furthermore, international journals require evidence of ethics review and informed consent and hence, the lack of attention to such research ethics practices might represent one reason to explain the under-representation of developing countries in the research literature [12].

Finally, our results might reflect investigators' lack of adequate or reinforced training in human subjects protection. It is unclear whether the results of this study demonstrate what might be an appropriate learning curve in investigators' awareness and educational capacity regarding research ethics practices, as this topic has only been introduced in developing countries during the last decade. We do not have data as to how our results compare with other WHO Regions. Nonetheless, the results emphasize the need to continue to raise the overall level of awareness and training among researchers regarding the importance of considering explicitly various ethical safeguards in the conduct of their research. Accordingly, we recommend that educational efforts be enhanced to emphasize the implications of research ethics on the value of protecting the welfare and rights of individuals who volunteer to participate in research studies. Presently, there are several existing programs that aim to help strengthen research ethics capacity in the EM Region. For example, the United Nations Educational, Scientific and Cultural Organization (UNESCO) has launched several recent programs aimed at strengthening research ethics in the Arab Region [13]. The Wellcome Trust in the United Kingdom provides support to build ethics capacity in resource-poor countries that have well-established research centers [14]. The Fogarty International Center of the U.S. National Institutes of Health has provided funding for the establishment of training programs in research ethics for individuals from the Middle East [15]. Based on the results of this study and other sources of data, the WHO is continuing national training initiatives for ethics in biomedical research in the different member countries of the EM Region. Finally, individual countries should adopt national research ethics regulations to ensure a consistent approach to the review of research. A robust human subject protection program that includes research ethics training and a strong system of ethics review can help ensure the public trust in research and enhance the research agenda in the developing world.
